# Design Features in Multiple Generations of Electronic Cigarette Atomizers

**DOI:** 10.3390/ijerph16162904

**Published:** 2019-08-14

**Authors:** Monique Williams, Prue Talbot

**Affiliations:** Department of Molecular, Cell, and Systems Biology, University of California, Riverside, CA 92521, USA

**Keywords:** electronic cigarette, e-cigarette, design features, atomizer, cig-a-like, clearomizer, mods

## Abstract

The design of electronic cigarette (EC) atomizing units has evolved since their introduction over 10 years ago. The purpose of this study was to evaluate atomizer design in ECs sold between 2011–2017. Atomizers from 34 brands representing three generations of ECs were dissected and photographed using a stereoscopic microscope. Five distinct atomizer design categories were identified in first generation products (cig-a-like/cartomizer) and three categories were found in the third generation. Atomizers in most cig-a-like ECs contained a filament, thick wire, wire joints, air-tube, wick, sheath, and fibers, while some later models lacked some of these components. Over time design changes included an increase in atomizer size; removal of solder joints between wires; removal of Polyfil fibers; and removal of the microprocessor from Vuse. In second and third generation ECs, the reservoirs and batteries were larger, and the atomizing units generally lacked a thick wire, fibers, and sheath. These data contribute to an understanding of atomizer design and show that there is no single design for ECs, which are continually evolving. The design of the atomizer is particularly important as it affects the performance of ECs and what transfers into the aerosol.

## 1. Introduction

Electronic cigarettes (ECs) are tobacco-free nicotine delivery devices that have gained world-wide popularity and have become a multi-billion dollar industry [1]. All ECs have three basic components: a battery, atomizer, and fluid reservoir, which stores the e-liquid [2,3]. There are several mechanical steps that take place to produce the aerosol. First, the user draws air through the mouthpiece, which activates an air-flow sensor, causing the filament in the atomizer to heat. The e-liquid is brought to the filament via capillary action created by the wick [4,5]. The heated filament vaporizes the e-fluid to produce a gas that condenses with water in the atmosphere to form an inhalable aerosol [4,5,6]. Some products lack an air flow sensor. In these, pressing a button closes a circuit that activates the battery, which in turn heats the filament [7]. The heating process is important as the temperature and components of the atomizer can influence the chemicals that transfer into the aerosols [8,9,10]. Some of these chemicals are toxic and could produce adverse health effects [11,12,13,14,15,16].

The characteristics and composition of the aerosol can be influenced by a number of factors, such as battery power level [8,13,16], topography [9,17,18,19], and one of the most important, atomizer design. For example, early models of ECs had tin solder joints that connected the filament to a thicker wire. In some brands, these solder joints were friable, and high concentrations of tin were found in their aerosols [20]. In the same brand, some samples had solder joints that were stable, and their aerosols had low concentrations of tin [20]. In other early brands of ECs, tin concentrations in aerosols were reduced by coating the thick wire with silver rather than tin, using stable tin solder joints outside of the atomizer, or joining wires by clamping or brazing rather than soldering [20,21]. These data demonstrate the feasibility of removing elements/metals from the aerosol by altering atomizer design. 

**Figure 1:**
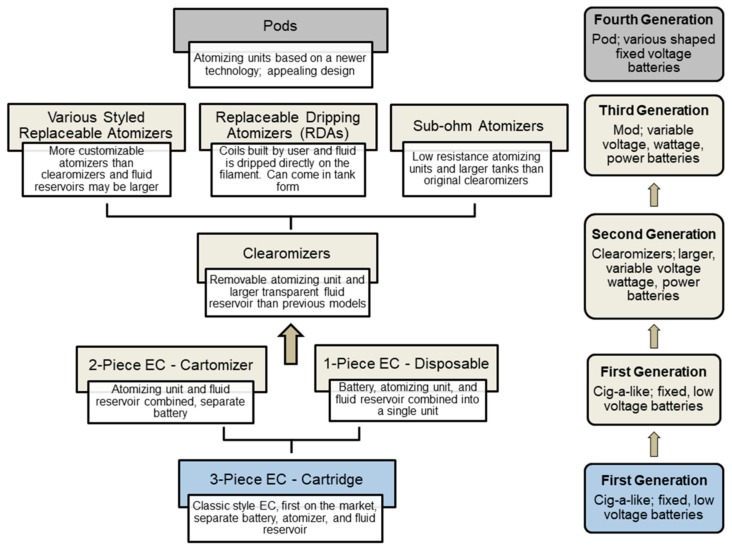
General characteristics of four generations of electronic cigarettes (ECs) and atomizing units. The boxes in the column on the right are terms used to describe the three generations of ECs [4]. These terms are based on the external appearance of the EC (cig-a-like and clearomizer) and on whether it is modified (Mod). Each box gives the generation number and the main features of the battery for each generation. The boxes on the left describe the atomizing units found in ECs of each generation. Each box is titled with the overall group classification name (e.g., “3-Piece EC”) followed by a description of the battery, atomizing unit, and fluid reservoir. Blue box = not included in this study; light brown boxes = included in the study; grey box = an emerging class of ECs not included in this study.

Since their introduction over 10 years ago, EC design has evolved in several ways. As a result various schemes have been introduced to characterize this evolution, and these can often be confusing [2,4,22,23]. For the purposes of this study, the scheme described in the recent report on ECs by the National Academy of Science, Engineering, and Medicine [4] will be used. This report recognized three generations of ECs: the cig-a-like (first generation), clearomizer (second generation), and mod (third generation) [4]. A fourth emerging generation, the pod, is not included in this study, but is rapidly gaining popularity [24]. The types of ECs used in this study are shown in Figure 1. The characteristics of each generation and their batteries are grouped in the boxes on the right and the atomizing units are grouped in the boxes on the left. Often generational classification schemes do not consider the evolution of atomizers, which have undergone a series of design changes in each generation. 

First generation ECs were designed to have the look and feel of a conventional cigarette and are often referred to as “cig-a-likes”, which come with fixed, low voltage batteries (Figure 1). The first generation cig-a-like atomizing units come in three versions: (1) the 3-piece style, which is the original EC, has a separate atomizing unit, battery, and fluid reservoir [25]; (2) the 2-piece style, in which the atomizing unit and fluid reservoir are combined, and the battery is separate; and (3) the 1-piece disposable, which combines the atomizing unit, fluid reservoir, and battery into a single unit (Figure 1, Figure S1) [25,26,27]. The original classic style ECs are no longer available. The 2-piece ECs are still widely sold on the Internet and in convenience stores, supermarkets, and gas stations [4,28]. In 2013, manufacturers created the 1-piece disposable EC, which was designed to be discarded after one use [26,29]. The 2- and 3-piece cig-a-like style ECs have batteries which can be recharged (with the exception of the disposable models) and prefilled low volume fluid reservoirs, which are not usually intended to be refilled (Figure 1). For some brands of the 2-piece EC, empty reservoirs can be purchased and filled by the consumer. 

Second generation ECs, known as “clearomizers”, often have larger variable voltage batteries, sometimes referred to as pen-style batteries (Figure 1) [27,30,31,32]. Second generation clearomizers have a removable atomizing unit that has a filament and comes encased in a shell that is screwed into the fluid reservoir and the battery. The clearomizers are transparent and have higher volume fluid reservoirs (or tanks) than cig-a-like style ECs (Figure 1). Clearomizers can be filled with any refill fluids that are currently available. 

Third generation ECs are known as “Mods”, which include modified batteries that allow the consumer to vary the voltage, wattage, and power, and some models come with added features, such as the ability to charge a cell phone (Figure 1). While some research groups have classified sub-ohm batteries into a “fourth” generation [22,23], the National Academy of Science, Engineering, and Medicine classification scheme was used in this study since sub-ohm batteries have variable voltage and wattage, which is characteristic of third generation ECs [4]. The atomizing units in the third generation come in three versions: various styled, replaceable dripping, and sub-ohm (Figure 1) [33]. These atomizing units have various shapes and coil composition. The fluid reservoirs typically disassemble to allow more customizability and may be larger than clearomizers (Figure 1). For the replaceable dripping atomizers (RDAs), the main characteristic is that the consumer builds their own filaments/coils and either the refill fluid is dripped directly onto the coils or the atomizer is encased in a fluid reservoir/tank (Figure 1). The sub-ohm atomizing units, which have low resistance and can be used at higher variable voltages and wattage, come prebuilt (Figure 1). 

The fourth generation of ECs, as classified in Figure 1, includes the pod-style that comes with fix voltage and various shaped batteries, such as USB or teardrop shapes (Figure 1) [24,34,35]. Since this generation is rapidly changing and has many new entries, it was not covered in this study. 

Atomizers are essential components of all ECs and their design and operation can affect what the ECs deliver to users, therefore it is important to understand how atomizers are built and their component parts. There have been several studies on the battery and reservoir design [2,22,23] and the atomizer design [7,20,21,36] of ECs, but no studies tracking EC atomizer designs as they have changed during the evolution of these products within or between brands. The purposes of this study were to: (1) evaluate the design of the atomizers in three generations of ECs over seven years; (2) compare this to the atomizer design of first generation disposable ECs [7]; and (3) determine how the design of atomizing units changed within a brand during product evolution. 

## 2. Materials and Methods 

### 2.1. Electronic Cigarette Selection

This study focuses on the design of atomizers in ECs that were purchased on the Internet between 2011–2017, were available nationwide (US), and were manufactured by both major tobacco companies (Mark Ten and Vuse) and independent manufacturers (e.g., South Beach Smoke and Tsunami). Brands were selected by searching “electronic cigarettes” on the Internet, and top brands in the search were purchased. In addition, many of the brands that were included in this study were used in previous performance testing studies [26,37,38]. 

First generation products that were studied included: BluCig and BluCig Plus (Lorillard Inc., Greensboro, NC, USA), Mark Ten and Mark Ten XL (Altria Group Inc., Richmond, VA, USA), V2 Cigs (VMR Products LLC, Miami, FL, USA), and Vuse and Vuse Vibe (Reynolds American Inc., Winston-Salem, NC, USA). Other brands used in the study were Crown 7 Imperial Hydro (Crown Seven Shop, Scottsdale, AZ, USA), Green Smoke (Green Smoke LLC, Richmond, VA, USA), Liberty Stix Eagle (Liberty Stix LLC, Cleveland, OH, USA), NJOY NPRO 2N1 (Sottera Inc., Scottsdale, AZ, USA), Safe Cig (The Safe Cig LLC, Los Angeles, CA, USA), Smoke 51 (Vapor Corp, Miami, FL, USA), Smoking Everywhere Platinum (Smoking Everywhere, Sunrise, FL, USA), and South Beach Smoke (South Beach Java LP, Wood Dale, IL, USA). Upon receipt, all ECs were inventoried and stored at room temperature. All EC cartomizers were tobacco flavored with “high” nicotine concentrations.

To study the design of the second and third generation ECs, five batteries, four tanks, and two RDAs were selected based on their popularity between 2014–2017. Popularity was established by speaking with clerks at a local vape shop near the University of California, Riverside (UCR) campus and mining information on leading refill fluid manufacturers’ websites. Product choices do not necessarily represent popularity in other regions of the country. The following EC batteries were used: Ego C-Twist (Joyetech Co., Shenzhen, China), iTaste MVP 2.0 (Innokin, Henzhen, China), Nemesis (Shenzhen HCIGAR Technology Co., Ltd., Shenzhen, China), iPV6X (Pioneer4you, Shenzhen iPV Vaping Technology Co., Shenzhen, Guangdong, China), and Smok Alien (Shenzhen IVPS Technology Co., Ltd., Shenzhen, China). The following tanks and RDAs were used: Kangertech Protank (Kangertech, Shenzhen, China), Aspire Nautilus tank (Aspire, Shenzhen, China), Kanger T3S tank (Kangertech, Shenzhen, China), Tsunami 2.4 (Tsunami Vapor Glass, Troy, MI, USA), Smok tank (Shenzhen IVPS Technology Co., Ltd., Shenzhen, China), and Clone RDA. Products were inventoried and stored at room temperature. All EC products used and their classifications can be found in Supplemental Table S1.

### 2.2. Dissections of EC Atomizer Components

All first generation cig-a-likes were cut below the battery-cartomizer interface to reveal the intact atomizing unit. The underlying fibers were removed using forceps, exposing the wires, the joints between the wires, air-tube, wick, and sheaths. For second and third generation clearomizer and mod-style ECs, the atomizing units were split where the filament was located, with the exception of the RDAs, which were solid units. The components of interest were dissected from each atomizing unit as described previously [20,36], and the following were recorded: the lab inventory letter code assigned to each unit, EC style, brand, year purchased, type of activation, flavor, nicotine concentration, presence of fibers, whether the Polyfil was centrifuged after dissection, the amount of fluid recovered upon centrifugation, fluid color, presence of a filament, thick wire, wick, air-tube, sheath, number of sheaths, wire-to-wire joints, integrity of the wire, condition of the joints and wick, and evidence of use before purchase. All dissections were photographed using a Canon SLR digital camera, and individual components were imaged using the Nikon SMZ 745 stereomicroscope. All dissections were done on unused products, except for NJOY NPRO 2N1 (2011), which had been used by us prior to dissection.

## 3. Results

### 3.1. Design and Anatomy of Cig-a-Like Style ECs

**Figure 2:**
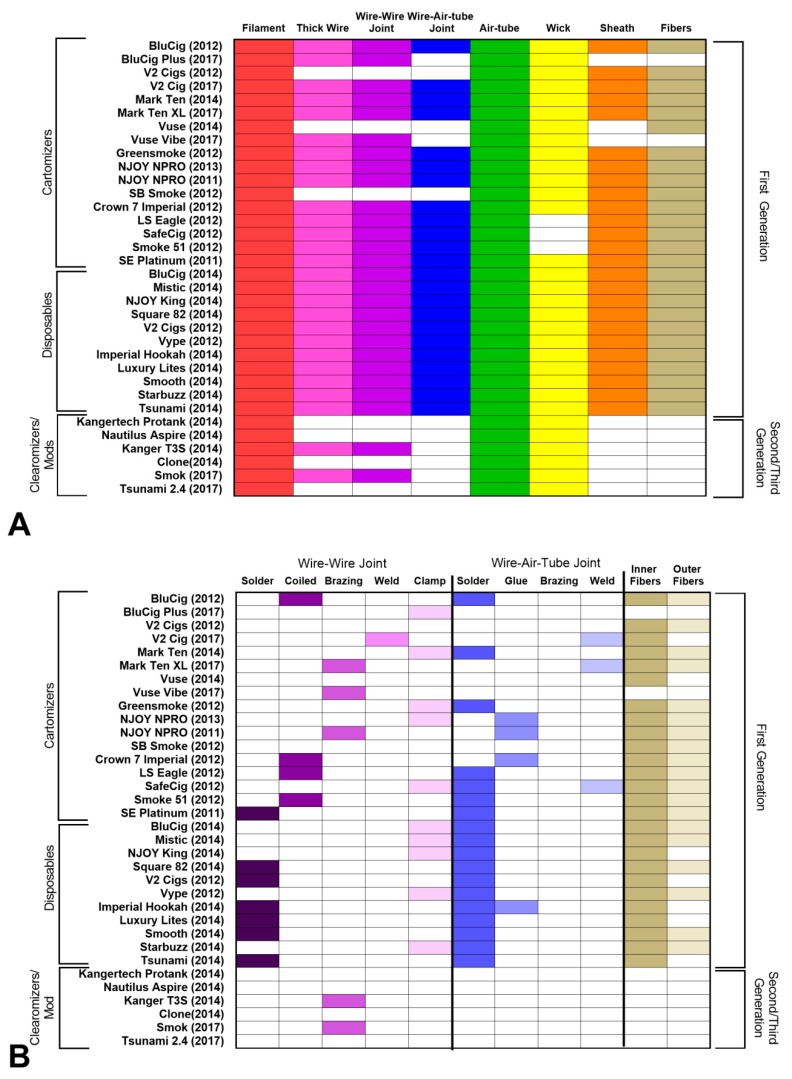
Components of the atomizing units across brands and generations of ECs. Tables show the presence or absence of an atomizing unit component in each EC. (**A**) Major components (filament, thick wire, wire-wire joint, wire-wire-tube joint, air-tube, wick, sheath, fibers) present in ECs. (**B**) Methods of joining components (wire–wire joint, wire–air-tube joint) and presence or absence of fiber types. Boxes in color = component is present, white boxes = the component is absent.

**Figure 3:**
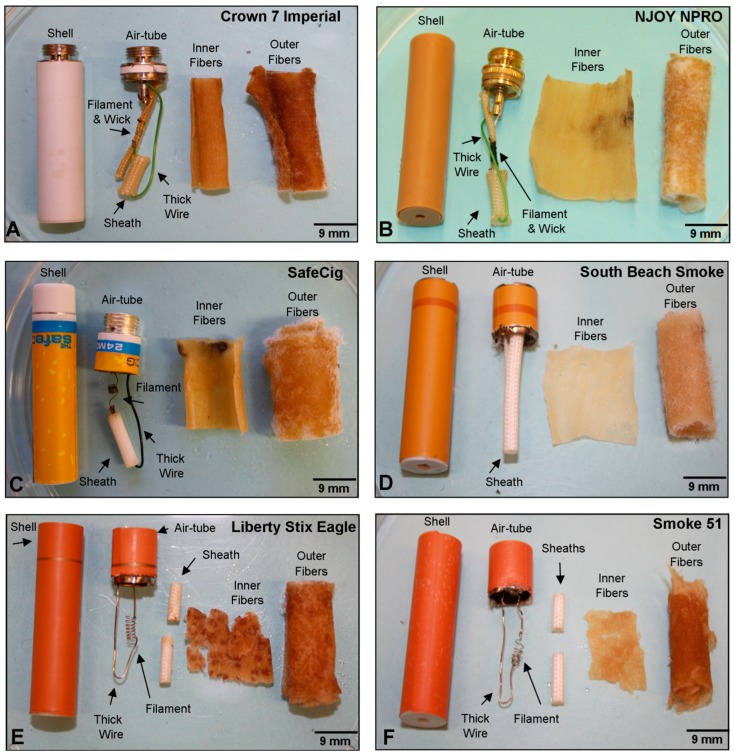
Anatomy of atomizers from cartomizer style ECs showing three different design categories. (**A**) Crown 7 Imperial, (**B**) NJOY NPRO, (**C**) SafeCig, (**D**) South Beach Smoke, (**E**) Liberty Stix Eagle, (**F**) Smoke 51. The shell, air-tube, filament, wick, sheath, thick wire, and inner and outer fibers are labeled in (**A**). Design category 1 (**A**–**C**), category 2 (**D**), and category 3 (**E**–**F**).

**Figure 4:**
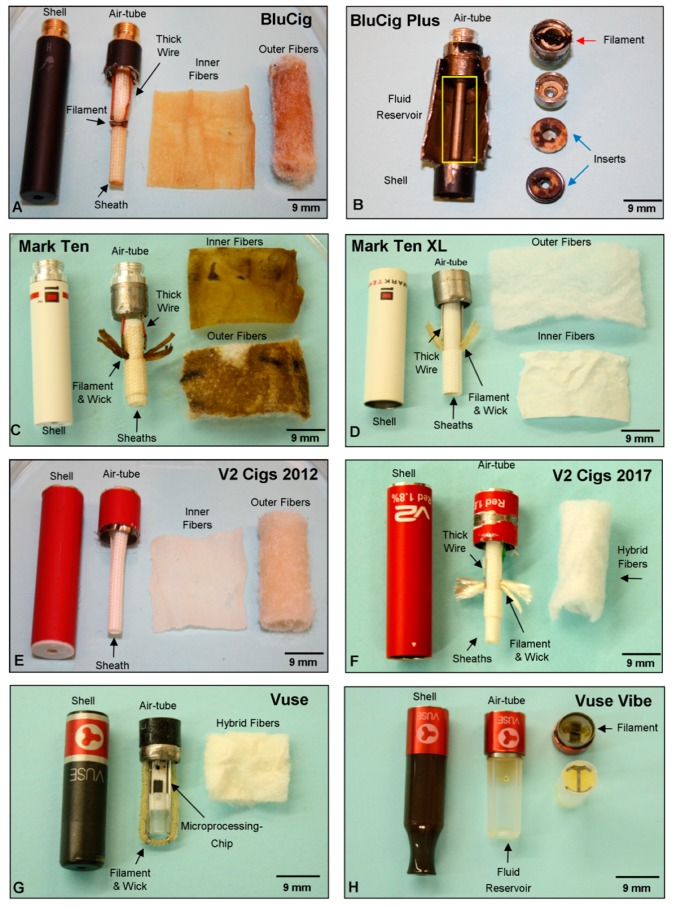
Comparison of atomizers from four brands of first generation cartomizer style ECs across different generations. The internal anatomy of (**A**) BluCig, (**B**) BluCig Plus, (**C**) Mark Ten, (**D**) Mark Ten XL, (**E**) V2 Cigs 2012, (**F**) V2 Cigs 2017, (**G**) Vuse, and (**H**) Vuse Vibe. Yellow box in (**B**) indicates the reservoir, red arrow in (**B**) indicates the filament, and the blue arrow in (**B**) indicates the inserts in the BluCig Plus. Design category four (**A**), and category five (**B**,**G**,**H**).

First generation (cig-a-like) cartomizer style ECs (Figure 2) were purchased between 2011 and 2017, and the internal design of the atomizers was compared (Figure 2, Figure 3 and Figure 4). All cartomizer style ECs contained a filament and an air-tube, and most contained a thick wire, joints between wires, a wick, sheath(s), and fibers (Figure 2A). Most brands had both inner and outer fibers, although a few had only a single fiber type that was a hybrid of the densely packed inner fibers and outer Polyfil (Figure 2B). When both wire types were present, most brands joined the wires via solder or a clamp; other methods of joining included coiling, brazing, and welding (Figure 2B). Solder was the dominant method of joining the thick wire to the air-tube (Figure 2B), with glue or welding being less frequently used methods. 

The atomizer design of the first generation cig-a-likes could be classified into five categories (Figure 3 and Figure 4). The first design category consisted of an insulated thick wire, coiled filament, solder joints between the wires, a wick, and two fiber types (densely packed inner fibers and loosely packed outer fibers) (Figure 3A–C). Within this category of atomizer design, the presence of a wick and the size and shape of the sheaths varied. In addition, one brand (NJOY NPRO) had a gold plated air-tube, and over the years shifted from having a plastic outer shell/mouthpiece to a metal outer shell (Figure 3B). Brands in this category were Smoking Everywhere Platinum, Crown 7 Imperial, NJOY NPRO 2N1 (2011, 2013), and SafeCig (Figure 3A–C) [20,36].

The second design category contained a wick, single filament, and a long sheath that extended the length of the cartomizer with two fiber types (Figure 3D). Two brands (South Beach Smoke, V2 Cigs 2012) had this internal design. The third design category was similar to the first category and consisted of un-insulated thick wires connected to the thin filament, two short sheaths, and two fiber types (Figure 3E,F). Unlike the category one cartomizer design, the inner fibers that wrapped around the atomizing unit were very delicate and easily shredded when dissected. Two brands, Liberty Stix Eagle and Smoke 51, had this internal design. 

The fourth design category was a hybrid of category one and two. It consisted of insulated thick wires, a coiled thin filament, wire joints, a wick, multiple long sheaths, and two fiber types, as seen in BluCig (Figure 4A). Unlike any other brands, this atomizer design contained more than one sheath: a long sheath that extended the length of the cartomizer, and a larger sheath that fit over the base of the long sheath, as seen in Mark Ten, Mark Ten XL, and V2 Cig 2017 (Figure 4C,D,F). One brand (Greensmoke) that contained this design differed by having three sheaths and only one fiber type that was not tightly packed together [20]. The last atomizer design category was found in BluCig Plus, Vuse, and Vuse Vibe. Each had its own independent design that was not similar to any other design category (Figure 4B,G,H). 

### 3.2. Evaluation of Atomizing Unit Design across Cartomizer Generations

To determine how atomizer designs changed over time, four brands of first generation cartomizer ECs were purchased between 2011–2017, and the atomizer designs were analyzed (Figure 4). Overall, cartomizers purchased in 2017 were larger in size than their predecessors to allow more storage of fluid, and for three of the four brands, the design was completely different than in the earlier models. 

In transitioning between BluCig and BluCig Plus, the manufacturer made four major changes to the atomizer design: (1) BluCig Plus eliminated the fibers and sheath, and used two donut-shaped inserts towards the end of the mouthpiece (blue arrow) (Figure 4A,B); (2) in place of fibers, BluCig Plus had a reservoir to store fluid (yellow box in Figure 4B) with a long metal tube that ran along the center of the cartomizer; (3) the filament was located between two metal shells with a ceramic-like cylinder inside (red arrow); and (4) the shell that housed the filament was connected to the metal tube in the reservoir. When the filament heated the metal tube, it aerosolized the fluid (Figure 4B). 

The Mark Ten XL was identical to the Mark Ten, except it was larger in size and the battery screwed into the cartomizer as opposed to the cartomizer screwing into the battery (Figure 4C,D). The Mark Ten XL was easier to operate on the smoking machine, although the reason for this is not obvious from its design. The V2 Cigs 2017 differed from the 2012 model, in that it had a thick wire, wire joints, double sheath (one extended the length of the cartomizer, and a smaller one just below the wick), and a single fiber type that was a combination of densely woven and Polyfil fibers (Figure 4E,F).

Vuse and Vuse Vibe were also different between generations (Figure 4G,H). The most striking differences in the Vuse Vibe were: (1) the filament was not held in place by a scaffold; (2) it did not have a micro-processing chip like the original Vuse; (3) the size of the battery and cartomizer was almost double that of the original; (4) Vibe contained five times as much e-liquid as the Vuse; (5) it lacked fibers; and (6) the wick in the Vuse Vibe was four times shorter than that in Vuse (Figure 4G,H). Like the BluCig Plus, the Vuse Vibe filament was closer to the battery (Figure 4B,H).

### 3.3. Design and Anatomy of Second Generation Clearomizer and Third Generation Mod-Style ECs

**Figure 5:**
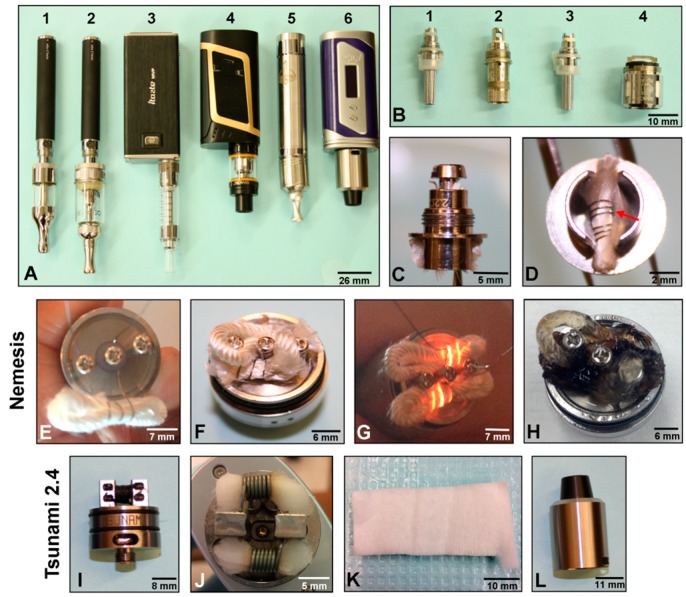
Comparison of batteries, reservoirs, and atomizing units in different models of second and third generation clearomizer/mod-style ECs. (**A**) Layout of all batteries and reservoirs used in the study: 1 (Ego C Twist, Kangertech Protank), 2 (Ego C Twist, Aspire Nautilus), 3 (iTaste MVP, Kanger T3S), 4 (Smok Alien, Smok), 5 (Nemesis, Clone), 6 (iPV6X, Tsunami 2.4). (**B**) Atomizing coils from left to right for 1 (Protank), 2 (Aspire), 3 (Kanger T3S), 4 (Smok). (**C**) Profile of top of the atomizing coil from Protank. (**D**) The wick and filament (red arrow) from Protank. (**E**) Partially built coil from Clone RDA. (**F**) Fully built Clone atomizer with two coils and wicks. (**G**) Testing the coils were properly built in the Clone atomizer. (**H**) Appearance of the coils from Clone atomizer following 60 puffs. (**I**) Side profile of the Tsunami atomizer. (**J**) Fully built Tsunami atomizer with wicks. (**K**) Detail of the wick for the Tsunami atomizer. (**L**) Cap for covering the Tsunami atomizer.

The external appearance of the batteries, reservoirs, and atomizing coils are presented in Figure 5. The batteries and reservoirs varied in size and design (Figure 5A). The batteries for the clearomizer/mod-style ECs were all significantly larger than those of the cig-a-like EC models. The atomizing units that heat the refill fluid stored in the reservoir of the clearomizer/mod-style ECs varied in size, design, and resistance (Figure 5B). The atomizing coils came either as two separate pieces that could be connected together or a single solid piece (Figure 5C–J). A side profile of the top of a clearomizer atomizing unit is shown in Figure 5C. The heating coil is located in the top piece, as shown in Figure 5D (red arrow). 

The atomizers in second and third generation ECs came in four designs: the clearomizer, customizable atomizer, the RDA, and the sub-ohm atomizer (Figure 1). The reservoirs consisted of either clearomizers, which do not come apart and are transparent so the consumer can see the fluid, or sub-ohm reservoirs, which have a larger capacity than the clearomizers and use low resistance coils (Figure 5A) [4,33]. The RDAs require the consumer to build their own coils and insert a wick [4,33]. Both of these types of atomizers/reservoirs came in different sizes, and some came apart to allow for more customizability (Figure S2A–C). In the newer models, the reservoirs were shorter and wider, and the atomizers were larger (Figure 5A,B). The RDAs allow the consumer to build the atomizer by choosing the wire and wick. Two RDAs were used in this study (Figure 5E–L). The Clone RDA, which requires two coiled wires and two folded wicks, is shown being assembled (Figure 5E) and after assembly with the coils and two wicks in place (Figure 5F). If the wires are connected properly, the coils will heat (Figure 5G). For the RDAs, the consumer drips refill fluid directly onto the heated coil, as seen for the Clone (Figure S2E), and the refill fluid changes color after use, becoming darker brown/black (Figure 5H). The Tsunami RDA is a newer style EC (Figure 5I) that uses a much thicker wire (Figure 5J) and a cotton wick (Figure 5K), which needed to be resaturated and changed frequently during use. All RDAs came with a case to cover the coils (e.g., Figure 5L), and these cases varied in size and shape. 

## 4. Discussion

The design features of atomizers were analyzed in ECs over a seven-year period. Previously published data on disposable ECs were also included in the comparison [7]. Results demonstrate that EC atomizer designs have evolved over time. Understanding design evolution is important in interpreting data on aerosol composition, a topic of recent interest [2,5,7,32,39]. Design analysis also helps understand how and why EC performance can vary among products. Most prior work on ECs has focused on battery features rather than atomizer design; nevertheless, information on both are valuable in making overall interpretations of data. The current study clearly shows that EC atomizer design varies among products and varies over time within product types, indicating that ECs are rapidly changing devices and that continual analysis of design is important. These data complement our recent study that characterized the elements/metals in atomizer components over a seven-year period [21]. 

Most atomizers in first generation ECs contained the same basic components; however, they differed sufficiently to enable their classification into five distinct design categories. The atomizer design in three of the four cartomizer style ECs (BluCig Plus, Vuse Vibe, V2 Cig) evolved during the study period. Within the cartomizer brands, the main design differences between the old and new models were: (1) increased fluid capacity in the newer cartomizers; (2) absence of Polyfil fibers in BluCig Plus; (3) changes in the methods used to join the filament and thick wire (e.g., brazing or clamping instead of solder, as seen in the Mark Ten); and (4) use of brazing or welding rather than soldering to join the thick wire to the air-tube, as seen in V2 Cigs. In the early models, the atomizers were delicate and easily damaged, which may account for the failure of some to be puffed and variations in performance within brands [26,37,38,40,41,42,43]. Within this group, there were design changes that appeared to provide protection for the filament and make the atomizer more robust. These changes included using a long sheath that covered the filament, moving the filament closer to the battery interface, and supporting the filament on a metal scaffold. 

The most striking differences in the overall design of the second and third generation ECs compared to first generation products were the increase in size of the fluid reservoirs and the larger sized batteries. The atomizing units per se in the second and third generation differed from first generation products in that they: (1) lacked a thick wire; (2) often had more than one filament; (3) usually encased the filament in a metal shell; (4) had no solder joints; (5) increased the mass of metal in the atomizers; and (6) lacked Polyfil or other types of fibers. While some metal components were absent in atomizers of third generation products, the overall amount of metal was greater. This coupled with the increase in battery power suggests that third generation products would release higher concentrations of metals into the aerosol than cig-a-like products. This idea is supported by observations on metal concentrations in disposable (first generation) versus tank style (third generation) ECs [7,31,32]. 

Differences between the second and third generation ECs were also apparent. Most clearomizers (e.g., Protank and Kanger T3S) had transparent reservoirs and consisted of the reservoir, atomizing unit, and the tank screw cap. In contrast, the Aspire, which is a third generation product, came apart completely and was much larger than the clearomizers. The newer third generation reservoirs (e.g., Smok), were smaller, wider, and contained larger atomizing units than the second generation products. The presence of two filaments in some third generation atomizers is a major design change, which allows more distributed heating and more production of aerosol [33]. However, aerosol production is also dependent on the type of battery, the voltage/wattage/power used, and the puff duration, which is highly variable among users [18,19]. The RDAs, which typically have two or more filaments, are much larger in size; however, a major disadvantage of the RDAs is that their operation requires the consumer to drip e-liquid onto the coils every few puffs to prevent “dry puffing” [22]. Users have reported that dripping creates larger clouds, enhances flavor, and gives stronger throat hits than other EC models [44]. Dripping devices have also been used with illicit drugs [45]. Some RDAs have tanks (referred to as RDTAs) that automate the dripping process, which helps prevent dry puffing and eliminates the need to frequently drip e-liquid onto the coils [22]. The Tsunami, one of the newer models studied, used a cotton rather than silicon wick. This may facilitate drawing fluid to the filament, but the cotton was labile and sometimes appeared charred, which could introduce new chemicals into the aerosols. Since RDAs are modifiable by users, they may perform differently within a brand. For example, if the screws that hold the filament in place are not tightened enough in the RDAs, the filament will not heat properly, and aerosol delivery will be negatively affected. 

It is important to understand atomizer design and composition in different EC generations, since elements in atomizers, such as nickel, chromium, and silicon, that may adversely affect health [5], can transfer to the aerosol during heating [7,20,21,31,32,39]. Second and third generation atomizers had fewer overall components than cig-a-like models (e.g., most lacked a thick wire, silicon sheath, and Polyfil fibers). Silicon is often the most abundant element in EC aerosols that are generated with products containing a silicon wick and sheath [7,36]. The elimination of the silicon sheath from second and third generation products may help reduce silicon concentrations in their EC aerosols. The thick wire found in first generation products is usually made of nickel or copper coated with either tin or silver [21], so its absence from second/third generation products could help reduce levels of these elements in aerosols. 

Another major change in atomizer design has been a reduction in the use of tin solder joints. In some early cartomizer models, such as Smoking Everywhere Platinum, manufacturers used tin solder to stabilize wire–wire and wire–air-tube joints [36]. While solder joints were not present between wires of BluCig, V2 Cigs, Mark Ten, or Vuse, or in any of the second and third generation atomizing units, they were used to join wires in most disposable brands [7,20]. Solder joints were also present between the air-tube and thick wire in most cartomizer and disposable products, while some had thick wires that were joined to the air-tube by brazing. These observations support the conclusion that there has been a manufacturing trend away from using tin solder joints between the filament and thick wire, but not between the thick wire and air-tube. When solder joints were observed in newer products, they generally appeared more stable than those observed previously in Smoking Everywhere Platinum [36]. The use of fewer tin solder joints and the elimination of tin solder between the filament and thick wire are important because they reduce tin in the aerosol [20]. Since long-term inhalation of tin can cause stannosis and pneumoconiosis [20,46], these diseases would not be as likely to occur when newer products are used. Also, some tin solder joints have contained lead [7,21,36], which would be a health concern as its inhalation could eventually cause damage to the nervous system and kidneys [47]. 

The performance of ECs can be affected by atomizer design. The thick-to-thin wire connection within the atomizing unit is very important in the performance of the ECs. Smoking Everyone Platinum joined the thick and thin wires with friable solder joints [36], and this brand often performed poorly when tested on a smoking machine [37]. In contrast, other brands (e.g., disposable V2 Cigs and Smooth) with stable solder joints often produced robust aerosols [7]. Since most brands, except disposables, have moved away from solder joints between the thick and thin wires, friable solder should not be a problem in newer models. EC brands in which the thin and thick wires were joined by brazing (e.g., NJOY NPRO 2011), clamps (e.g., SafeCig, Greensmoke and disposables such as BluCig, NJOY King, Starbuzz), or only contained a single wire/filament (e.g., South Beach Smoke, V2 Cig 2012, Vuse), all produced robust puffs when the devices were used on a smoking machine [26,37,38]. However, brands that joined the thick wire and filament via coiling the wires (Crown 7 Imperial, Liberty Stix Eagle, Smoke 51) did not produce much aerosol [37,38], indicating this is not an effective method of joining EC wires. Coiling was not used in the newer cartomizer products. Atomizer performance is also influenced by the batteries. The more powerful batteries and additional coils that accompany second and third generation ECs can produce larger amounts of aerosol, which are attractive to some users [33]. 

As the design features of atomizers have evolved, the batteries have changed with them. Cig-a-like ECs generally had low voltage batteries which did not change much with atomizer evolution. However, second and third generation ECs had larger, more powerful batteries with various options. The original second generation batteries were often pen style and some allowed variable voltage and wattage [48]. Subsequently, mods gave consumers more controllable battery features [48]. Most recently, sub-ohm batteries allow complete control of power, wattage, and voltage [48], and in Southern California are currently the most widely sold battery for use with third generation ECs. The increase in battery size was accompanied by an increase in atomizer size and mass of metal. The combination of the more powerful battery and larger atomizer enables users to take larger puffs and create larger exhaled clouds of aerosols [33]. These increases in battery power are important as they can also affect the output of the atomizers [9,10] and may result in a greater transfer of particles [9,23], metals [31,32], chemicals, such as nicotine [9,17], and toxicants, such as carbonyls and aldehydes [8,10,16,49], to aerosols. In addition, as battery voltage/power increases, new potentially toxic by-products can form from the EC liquids [10,13,40,50,51].

The reservoirs associated with the atomizers were different in each generation of ECs. In the cig-a-like models, there was variation between brands. The cartomizer and the disposable fluid reservoirs were generally similar in size. However, the newer cartomizer reservoirs were larger (e.g., the Vuse Vibe reservoir contained five times as much fluid as the Vuse, and the Mark Ten XL was longer and wider than its predecessor the Mark Ten). In contrast to cig-a-like models, second and third generation reservoirs were significantly larger and held from 2–5 mL of fluid, with the exception of the RDAs which held ~1 mL. This major design change in reservoir size is beneficial and cost effective to the consumer since they do not have to frequently refill or replace cartomizers or disposable devices. However, in the large reservoirs, fluid may not be refreshed as frequently and could acquire toxicants through repeated use [32]. In the second and third generation products, fluids darkened with use and black deposits accumulated on the filament and wick with repeated use. The black residue is likely charred organic material from the fluid. As the atomizers/reservoirs have evolved, fluid capacity has increased, which would tend to reduce the probability of dry puffing.

All of the EC styles in this study are eventually discarded and enter the environment. It is not currently clear how users are disposing of ECs and if they are entering landfills or recycling stations. In landfills, the battery chemicals and fluid residues in atomizers/reservoirs as well as the elements in the atomizers are likely to leach into the environment, and the impact of such leachates should be investigated.

## 5. Conclusions

ECs are evolving products that have undergone significant design changes between 2011 and 2017. Although the atomizer designs in the 2011 cartomizer products were similar, five distinct atomizers design categories were identified. Over time these designs changed with major differences being an increase in atomizer size, removal of solder joints between the wires, removal of Polyfil fibers, and removal of the microprocessor from Vuse. In contrast to cartomizers, second and third generation ECs had larger atomizing units, often with fewer components, larger reservoirs, and larger batteries. These data clearly show that there is no single design for ECs and that numerous designs have evolved over a seven-year period and will likely continue to evolve. The design of the atomizer in particular is important as it affects aerosol formation as well as what transfers into the aerosol. While this study contributes to a basic understanding of atomizer design, it is important in the future to track designs, determine how they evolve, and how they affect data. The design data in the current study will help focus attention on those atomizer components that are generally found across all types of EC products, are most prevalent in EC atomizers, are likely to affect aerosol composition, and are likely to enter the environment following EC disposal.

## Supplementary Materials

The following are available online at https://www.mdpi.com/1660-4601/16/16/2904/s1, Figure S1: Layout of each generation of EC. Figure S2: Anatomy of various tank style EC. Table S1: List of EC products used in the study, style, description, battery type, and generation.
