# Supplementary material for: Design Features in Multiple Generations of Electronic Cigarette Atomizers
Source: Int J Environ Res Public Health. 2019 Aug 14;16(16):2904. doi: 10.3390/ijerph16162904 (PMC6720609; doi:10.3390/ijerph16162904)
Supplement: Supplementary file 1 [file ijerph-16-02904-s001.pdf]

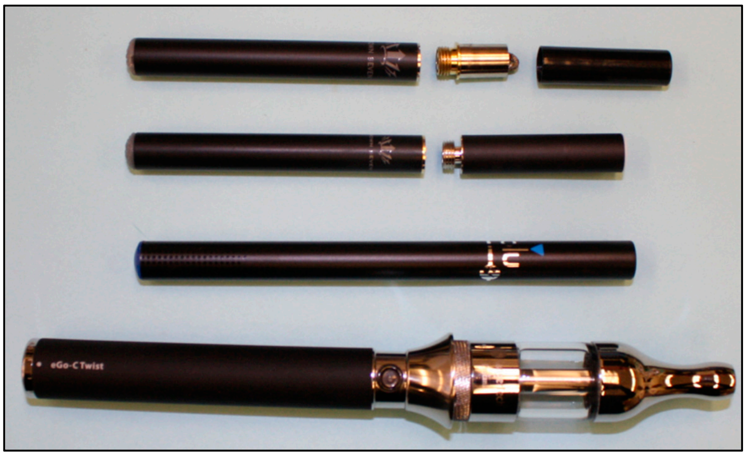

Figure S1. Layout of each generation of EC. (Top to bottom) 3- piece cartridge style, 2-piece cartomizer style, 1-piece disposable style, and tank style.

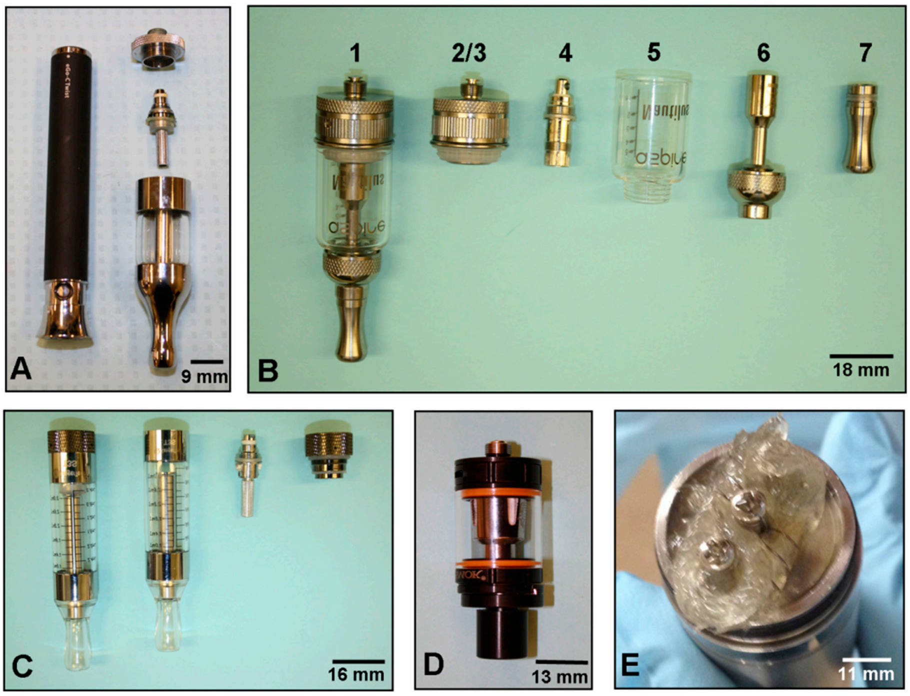

Figure S2. Anatomy of various tank style EC. A. Disassembled Kangertech Protank with associated coil and bottom hardware. B. Disassembled Aspire Nautilus tank and associated components: 1= fully assembled Aspire tank, 2/3 = bottom hardware and air-flow adjustment ring, 4 = replaceable atomizer/coil, 5 = Pyrex glass tank, 6 = upper hardware, 7 = drip tip/ mouthpiece. C. Disassembled Kanger T3S tank with associated coil and bottom hardware. D. Detail of fully assembled Smok tank. E. Anatomy of an unused Clone atomizer saturated with refill fluid.

Supplemental Table 1: List of EC products used in study

Brand	EC style	Description of EC	Battery Type	Generation
BluCig (2012)	Cartomizer	Cig-a-like	Fixed low voltage	First
BluCig Plus (2017)	Cartomizer	Cig-a-like	Fixed low voltage	First
V2 Cigs (2012)	Cartomizer	Cig-a-like	Fixed low voltage	First
V2 Cig (2017)	Cartomizer	Cig-a-like	Fixed low voltage	First
Mark Ten (2014)	Cartomizer	Cig-a-like	Fixed low voltage	First
Mark Ten XL (2017)	Cartomizer	Cig-a-like	Fixed low voltage	First
Vuse (2014)	Cartomizer	Cig-a-like	Fixed low voltage	First
Vuse Vibe (2017)	Cartomizer	Cig-a-like	Fixed low voltage	First
Greensmoke (2012)	Cartomizer	Cig-a-like	Fixed low voltage	First
NJOY NPRO (2013)	Cartomizer	Cig-a-like	Fixed low voltage	First
NJOY NPRO (2011)	Cartomizer	Cig-a-like	Fixed low voltage	First
SB Smoke (2012)	Cartomizer	Cig-a-like	Fixed low voltage	First
Crown 7 Imperial (2012)	Cartomizer	Cig-a-like	Fixed low voltage	First
LS Eagle (2012)	Cartomizer	Cig-a-like	Fixed low voltage	First
SafeCig (2012)	Cartomizer	Cig-a-like	Fixed low voltage	First
Smoke 51 (2012)	Cartomizer	Cig-a-like	Fixed low voltage	First
SE Platinum (2011)	Cartomizer	Cig-a-like	Fixed low voltage	First
BluCig (2014)	Disposable	Cig-a-like	Fixed low voltage	First
Mistic (2014)	Disposable	Cig-a-like	Fixed low voltage	First
NJOY King (2014)	Disposable	Cig-a-like	Fixed low voltage	First
Square 82 (2014)	Disposable	Cig-a-like	Fixed low voltage	First
V2 Cigs (2012)	Disposable	Cig-a-like	Fixed low voltage	First
Vype (2012)	Disposable	Cig-a-like	Fixed low voltage	First
Imperial Hookah (2014)	Disposable	Cig-a-like	Fixed low voltage	First
Luxury Lites (2014)	Disposable	Cig-a-like	Fixed low voltage	First
Smooth (2014)	Disposable	Cig-a-like	Fixed low voltage	First
Starbuzz (2014)	Disposable	Cig-a-like	Fixed low voltage	First
Tsunami (2014)	Disposable	Cig-a-like	Fixed low voltage	First
Kangertech Protank (2014)	Tank	Clearomizer	Variable voltage pen style	Second
Nautilus Aspire (2014)	Tank	Hybrid Clearomizer/Mod	Variable voltage en pen style	Second/Third
Kanger T3S (2014)	Tank	Hybrid Clearomizer/Mod	Variable voltage, wattage, power	Second/Third
Clone(2014)	Tank	Mod/RDA	Fixed voltage	Third
Smok (2017)	Tank	Mod/Sub-ohm	Variable voltage, wattage, power	Third
Tsunami 2.4 (2017)	Tank	Mod/RDA	Variable voltage, wattage, power	Third
